# Study of a spherical phantom for Gamma knife dosimetry

**DOI:** 10.1120/jacmp.v11i2.3130

**Published:** 2010-04-17

**Authors:** Dengsong Zhu, Carlos Austerlitz, Sidi Benhabib, Helvecio Mota, Ron R. Allison, Diana Campos

**Affiliations:** ^1^ Q.E.D. Medical Physics, Inc Lebanon TN USA; ^2^ Radiation Oncology Department The Brody School of Medicine at East Carolina University Greenville NC USA; ^3^ Carolinas Medical Center NorthEast – Radiation Oncology Concord NC USA; ^4^ Clinica Diana Campos Recife PE Brazil

**Keywords:** gamma knife, Penelope, Monte Carlo, water phantom, dosimetry, absolute calibration

## Abstract

Four 16 cm diameter spherical phantoms were modeled in this study: a homogenous water phantom, and three water phantoms with 1 cm thick shell each made of different materials (PMMA, Plastic Water™ and polystyrene). The PENELOPE Monte Carlo code was utilized to simulate photon beams from the Leksell Gamma Knife (LGK) unit and to determine absorbed dose to water $\left(D_{w}\right)$ from a single 18 mm beam delivered to each phantom. A score spherical volume of 0.007 cm^3^ was used to simulate the dimensions of the sensitive volume of the Exradin A‐16 ionization chamber, in the center of the phantom. In conclusion, the PMMA shell filled with water required a small correction for the determination of the absorbed dose, while remaining within the statistical uncertainty of the calculations (±0.71). Plastic Water™ and polystyrene shells can be used without correction. There is a potential advantage to measuring the 4 mm helmet output using these spherical water phantoms.

PACS numbers: 87.10.Rt, 87.50.cm

## I. INTRODUCTION

The Leksell Gamma Knife (LGK) is a complete system for radiosurgery marketed worldwide by Elekta. The effective non‐invasive treatment is made by 201 Cobalt‐60 (Co60) beams that have sufficient penetration to reach even the most deep‐seated tumors in the brain. The treatment is achieved by delivering prescribed doses (shots) of radiation, in compliance with a pre‐prepared treatment plan to the exact site of the intracranial target. The tissue in the target is thus treated by radiation while sparing surrounding tissue.^(1)^ It provides a low morbidity and effective alternative to conventional surgery.^(2)^ ^,^ ^(3)^ A spherical polystyrene phantom 160 mm in diameter furnished by Elekta is usually used in dosimetry, according to the American Association of Physicists in Medicine (AAPM) Task Group 21 Protocol.^(^ ^4^ ^–^ ^10^ ^)^

Currently, the worldwide trend in radiation dosimetry is to standardize absorbed dose to water $\left(D_{w}\right)$ as measured by a water phantom,^(11)^ and a new AAPM TG 51 protocol has been instituted for water calibration.^(12)^ It provides the possibility of reducing the uncertainty in the dosimetry of radiotherapy beams. Recently, a thimble‐shaped calibration water phantom, with a 2 mm plastic wall was designed for LGK calibration in water.^(13)^ This method avoids dosimetric uncertainties resulting from the composition of the traditional polystyrene phantom construction materials. In this work, four 16 cm diameter spherical phantoms were modeled: a homogenous water phantom, and three water phantoms with different 1 cm thick materials. The PENELOPE Monte Carlo code was utilized to simulate photon beams from the LGK unit and to determine $D_{w}$ from a single 18 mm beam delivered to center of each of the phantoms.

## II. MATERIALS AND METHODS

For the LGK Model B and Model C, the dose is delivered with 201 Cobalt‐60 (Co60) sources that are distributed on the surface of a hemisphere with a radius of about 400 mm, such that the beams are collimated to a common focal point (isocenter). Sources are distributed along five parallel circles separated from each other by an angle of 7.5°. Each one of the Co60 sources consists of 20 cylindrical Co60 pellets 1 mm in diameter and 1 mm in length. According to a simplified model,^(14)^ ^,^ ^(15)^ the active core can be considered as a unique cylindrical source of 1 mm diameter and 20 mm height made of Co60. The sources are encapsulated in 303 series stainless steel (C 1.0%, Mn 2.0%, P 0.045%, S 0.03%, Si 1.0%, Cr 18.0%, Ni 9.0%, Fe 69.8%) with welded closures. The dimensions of the source capsule are 7 mm in diameter and 33 mm long. Each beam channel consists of a 65 mm long tungsten cylinder with a radius of 2 mm in a precollimator followed by a 92.5 mm lead cone collimator and ends in a 60 mm interchangeable tungsten collimator helmet. The available sizes of the interchangeable collimator helmet are 4, 8, 14 and 18 mm.

**Figure 1:**
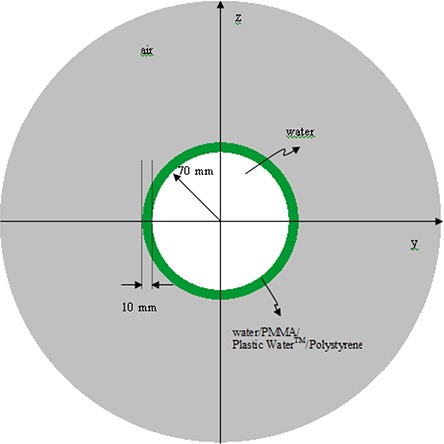
Schematic diagram of the spherical water phantom.

Four 16 cm diameter spherical phantoms were modeled: a homogenous water phantom and three water phantoms with 1 cm thick shells made of different materials. The shell materials were PMMA, Plastic Water™ and polystyrene. Table 1 gives the physical characteristics and composition of each material. The center coincides with the isocenter of the LGK unit. The schematic diagram of the spherical water phantom is shown in Fig. 1. Idealized experiments were performed using the PENELOPE Monte Carlo code for 18 mm collimator helmet to obtain the single‐beam profiles, isodose distribution, and the percent deviation in $D_{w}$ at the center of the phantoms.

**Table 1:** Composition (in weight percent fraction) and densities of PMMA, Plastic Water™ and polystyrene.

	*PMMA* ^a^	*Plastic Water™* ^b^	*Polystyrene* ^a^
H	8.05	9.25	7.7
C	59.98	62.82	92.3
N		1.00	
O	31.96	17.94	
Cl		0.96	
Ca		7.95	
Br		0.03	
Density $\left(g\;{\text{cm}}^{-3}\right)$	1.190	1.014	1.060

a As given by Penelope.

b As given by Tello et al,^(16)^ its density was generated by Penelope according to its composition.

The PENELOPE Monte Carlo code implements advance electron, positron and photon transport algorithms. It is suitable for simulating problems relevant to stereotactic radiosurgery.^(17)^ ^,^ ^(18)^ A detailed description on the structure of PENELOPE can be found in Salvat et al.^(19)^ During simulation, source capsule, precollimator and cone collimator (part I), collimator helmet (part II) and spherical water phantom (part III) were simulated separately. Two phase‐space files (PSFI and PSFII) were obtained during the simulation of part I and part II, respectively. PSFI contained all the particles scored at the plane below the inner surface of the tube collimator. PSFII contained all the particles scored at the plane below the inner surface of the final collimator helmet. The simulation was controlled by means of several parameters: $C_{1}$, $C_{2}$, $W_{\text{cc}}$ and $W_{\text{cr}}$, $E_{\text{abs}}$ (electron $e^{-}/e^{+}/\text{photon}$), as well as DSMAX. The first two refer to elastic collisions. $C_{1}$ represents an average angular deflection produced by multiple elastic scattering along a path length equal to the mean free path between hard elastic events. $C_{2}$ defines the maximum average fractional energy loss between consecutive hard elastic events. $W_{\text{cc}}$ and $W_{\text{cr}}$ are energy cutoff values to separate hard and soft events. The inelastic collisions with energy loss $W<W_{\text{cc}}$ and emission of bremsstrahlung photons with $W<W_{\text{cr}}$ are considered as soft stopping interactions in simulation. $E_{\text{abs}}$ (electron $e^{-}/e^{+}/\text{photon}$) is the absorption energy, when the energy becomes smaller than a given energy and particles are assumed to be effectively stopped and absorbed in the medium. The input parameter DSMAX defines the maximum allowed step length for $e^{-}/e^{+}$ for photons. In each simulation of part I, II and III, $C_{1}$ and $C_{2}$ are 0.1; $W_{\text{cc}}$ and $W_{\text{cr}}$ are 10 keV; $E_{\text{abs}}$ (electron $e^{-}/e^{+}/\text{photon}$) is 10 keV. DSMAX is given a value of the order of one‐tenth of the “thickness” of that body. In the simulation of part I, a total of $6.7\times 10^{7}$ histories were followed and the initial directions were sampled in a cone with semi‐aperture angle 10° toward the isocenter to get PSFI. In the simulation of part II, PSFI was used with a splitting factor 20 to obtain PSFII. In the simulation of part III, PSFII was used with a splitting factor 50. The statistical uncertainty of the Monte Carlo estimate is determined by the Eq.1 and Eq. 2:^(19)^

(1) $$\sigma_{Q}=\sqrt{\frac{1}{N\left[\frac{1}{N}\sum_{i=1}^{N}q_{i}^{2}-{\bar{Q}}^{2}\right]}}$$

(2) $$\bar{Q}=\frac{1}{N}\sum_{i=1}^{N}q_{i}$$

where *N* is a large number of simulated histories, $q_{1}$ is a random value scored by all particles of the $i_{th}$ history, and is an average score of *N*. The statistical uncertainties given throughout this paper correspond to $i_{th}$.

## III. RESULTS & DISCUSSION

**Figure 2:**
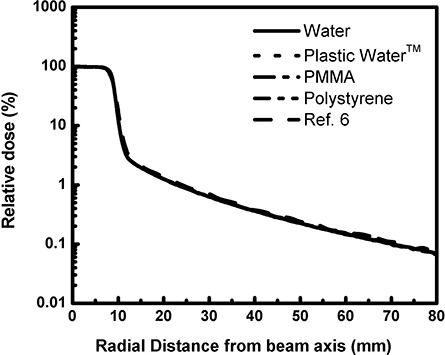
Comparison of single‐beam dose profile in the center of the spherical water phantoms with the different shell materials for the 18 mm collimator helmet.

Figure 2 shows the single‐beam dose profiles of three spherical phantoms for the 18 mm collimator helmet of the LGK unit, which were normalized in homogenous water at the region of dose maximum. The solid, dot, dash‐dot and dash‐dot‐dot lines correspond to the homogenous water phantom, water phantoms with 1cm Plastic Water™ shell, PMMA shell and polystyrene shell, respectively. The dash curve shows the single‐beam dose profile simulated by another Monte Carlo code, EGS4 for the 18 mm collimator helmet.^(6)^ In the study by Chung et al., the single‐beam dose profile was normalized with Elekta profiles at the region of dose maximum; the agreement is very good. Wu et al.^(20)^ reported that the dimension of the 50% isodose or full‐with‐ at‐ half‐ maximum (FWHM) of 18 mm collimator helmet equals 18 mm. As shown in Fig.2, our Monte Carlo results were consistent with those in the Wu et al. study.

**Figure 3:**
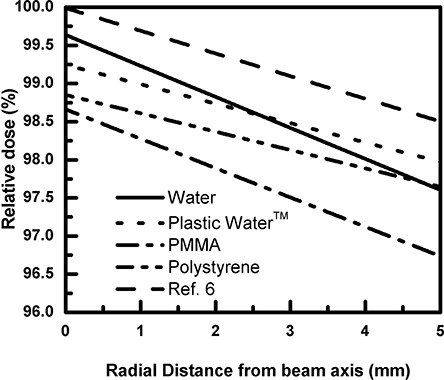
Isodose distribution in the center of the spherical water phantoms with the radial distance varying from 0 to 5 mm.

The curves in Fig. 3 were obtained by fitting the values of the relative dose for each material in a radial distance varying from 0 to 5 mm. Even though there was a relatively large uncertainty in the MC calculation, these results showed not only the expected relationship among the absorbed dose with these materials and their mass energy attenuation coefficient, but also the sensitivity of the modeling technique. Based only on the value of the energy absorption coefficient by the National Institute of Standards and Technology (NIST), a correction of about 0.2% and 0.8% were found for polystyrene and for PMMA, respectively, which were very close to that performed in this work. Nevertheless, the calculations performed in this work served to predict, in a more accurate fashion, the required amount of correction which was needed to compensate for the thickness of the shell material – rather than water, which was used in a spherical water phantom. Measurements performed with a 1 cm and 0.5 cm PMMA shell thickness are in progress and will be published.

**Figure 4:**
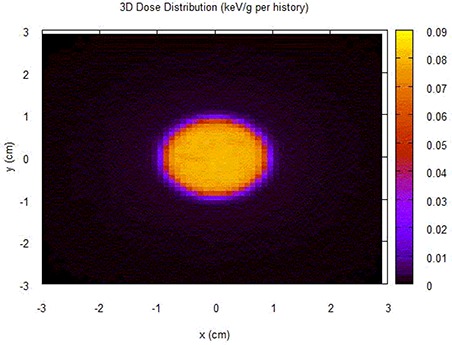
Isodose distribution in the center of the spherical water phantoms with the PMMA material for 18 mm collimator helmet. With the different shell materials, there is no observable difference.

Figure 4 shows the isodose distribution in the center of the spherical water phantom with the PMMA material for 18 mm collimator helmet. With the different shell materials, there is no observable difference.

The Exradin A‐16 chamber of collecting volume of 0.007 cm^3^ is used in the AAPM TG‐21 calibration of the LGK unit.^(9)^ A score spherical volume of 0.007 cm^3^ was used to simulate the dimensions of the sensitive volume of the Exradin A‐16 ionization chamber, in the center of the phantom. Within the statistical uncertainty of the modeling prediction (±0.71%), Table 2 gives the attenuation factor of the shell material in the $D_{w}$ in the center of the water phantom. Within the statistical uncertainty of the modeling prediction, the following percent deviations were found in $D_{w}$ in the center of the water phantom: 0.2%, 0.7%, and 1.4% as compared with homogenous water phantom for water phantom with Plastic Water™, polystyrene and PMMA shells, respectively.

**Table 2:** Attenuation factor of the shell material in the dose to water $\left(D_{w}\right)$ in the center of the water phantom; $D_{w}$ normalized to the homogenous water phantom. The statistical uncertainty of each simulation was ±0.71%.

*Shell Material, 1 cm thick*	$D_{w}$	*Attenuation Factor*
Water	1.000	N/A
PMMA	0.986	1.4%
Plastic Water™	0.998	0.2%
Polystyrene	0.993	0.7%

**Figure 5:**
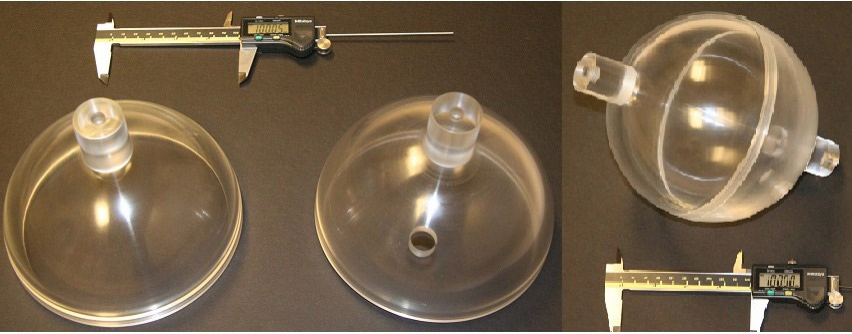
A spherical water phantom with 1 cm thick PMMA shell.

The spherical water phantom with 1 cm thick shell can be easily machined. A spherical water phantom with 1 cm thick PMMA shell made of two hemispheres and with a diameter of 16 cm was designed and built (Fig. 5). It can be positioned at the mechanical center of LGK and can be rotated in one plane. At the center of the phantom, either an ionization chamber or films can be placed with good accuracy, due to their geometric construction.

It is difficult to measure output factor of 4 mm helmet using ionization chambers due to their size. Film was used to measure the 4 mm helmet output factor for the LGK using a spherical polystyrene phantom 16 cm in diameter.^(21)^ ^,^ ^(22)^ Butson et al.^(23)^ reported that water would be the optimal medium for use in film dosimetry. The effects of water on the film are minimal because water could remove any spurious results caused by imperfect film‐phantom contact during parallel film exposure which could occur with the use of solid phantom. The film can be located at the center of our designed spherical water phantom. There is a potential advantage to measure the 4 mm helmet output using our spherical water phantom using film dosimetry instead of the ionization chamber.

## IV. CONCLUSIONS

In this work, four 16 cm diameter spherical phantoms were modeled: a homogenous water phantom, and three water phantoms with 1cm thick shells of different materials.

The PENELOPE Monte Carlo code was utilized to simulate photon beams from the LGK unit and to determine $D_{w}$ in their center from a single 18 mm beam delivered to each phantoms. A spherical volume of 0.007 cm^3^ was used to simulate the dimensions of the sensitive volume of the Exradin A‐16 ionization chamber in the center of the phantom.

The PMMA shell filled with water required a small correction for the determination of the absorbed dose, while remaining within the uncertainty of the calculations (±0.71). The Plastic Water™ and polystyrene shells can be used without correction. There is a potential advantage to measuring the 4 mm helmet output using these spherical water phantoms.

## ACKNOWLEDGEMENTS

This work was partially funded by the Brody Brothers Endowment Fund. The author Dengsong Zhu would like to acknowledge Dr. Shifeng Chen, from the Department of Radiation Oncology of University of Nebraska Medical Center, for his advice on 3D dose distribution and Dr. Haibo Lin, from Department of Radiation Oncology of University of Pennsylvania, for helpful discussions.
